# Temperature dependent fracture properties of shape memory alloys: novel findings and a comprehensive model

**DOI:** 10.1038/s41598-016-0024-1

**Published:** 2016-12-21

**Authors:** Carmine Maletta, Emanuele Sgambitterra, Fabrizio Niccoli

**Affiliations:** Department of Mechanical, Energy and Management Engineering, University of Calabria, P. Bucci 44C, 87036 Rende (CS), Italy

## Abstract

Temperature dependent fracture properties of NiTi-based Shape Memory Alloys (SMAs), within the pseudoelastic regime, were analyzed. In particular, the effective Stress Intensity Factor (SIF) was estimated, at different values of the testing temperature, by a fitting of the William’s expansion series, based on Digital Image Correlation (DIC) measurements. It was found that temperature plays an important role on SIF and on critical fast fracture conditions. As a consequence, Linear Elastic Fracture Mechanics (LEFM) approaches are not suitable to predict fracture properties of SMAs, as they do not consider the effects of temperature. On the contrary, good agreements between DIC results and the predictions of an *ad-hoc* analytical model were observed. In fact, the model takes into account the whole thermo mechanical loading condition, including both mechanical load and temperature. Results revealed that crack tip stress-induced transformations do not represent a toughening effect and this is a completely novel result within the SMA community. Furthremore, it was demonstrated that the analytical model can be actually used to define a temperature independent fracture toughness parameter. Therefore, a new approach is proposed, based on the analytical model, where both mechanical load and temperature are considered as loading parameters in SIF computation.

## Introduction

Shape memory alloys (SMAs), and in particular the Nickel-Titanium binary system (NiTi), have been becoming more and more attractive in recent years for both engineering and medical fields^1–3^, due to their interesting functional properties, namely shape memory effect (SME) and pseudoelastic effect (PE)^4^. Thanks to these features, SMAs exhibit very large recoverable deformation capabilities either by temperature or stress variations. These properties are obtained through a thermal or a mechanical induced reversible phase transformation between a parent austenite phase (B2) and a product martensite one (B19′), the so-called thermally induced martensite (TIM) and stress induced martensite (SIM).

Extensive researches have been carried out in last years, to better understand the relations between phase transition phenomena, occurring at the micro scale, and their thermo-mechanical response at the macro scale, with the aim of defining proper design criteria. In fact, standard procedures/methods based on classic solid mechanics theories cannot be directly applied to SMAs, due to their complex thermo-mechanical constitutive response associated with TIM and SIM. This is one of the main reasons why the use of SMAs is nowadays limited to special niche applications, mostly in the fields of medicine^3^, aeronautical and aerospace engineering etc. ref. 2. In these applications, design is usually based on empirical approaches, *i.e.* complex and expensive *ad-hoc* experimental campaigns are carried out for model development/calibration as well as for design assessment. In fact, the constitutive models for SMAs, implemented in commercial Finite Element (FE) software codes, are based on a large number of material parameters. Only a few of these parameters are measurable physical quantities, while other ones are purely phenomenological factors, which should be tuned to fit numerical results with experimental response.

The design task becomes even more complex when dealing with SMA components subjected to variable thermo-mechanical loading conditions. In fact, many aspects related with the evolution of both functional and mechanical properties under fatigue loadings, *i.e.* the loss of strain recovery capabilities as well as the crack formation and propagation mechanisms, are still unknown. This is of major concern because SMAs are today mostly used in critical biomedical applications, where they are employed for the realization cardiovascular stents, embolic protection filters etc. Fatigue failures in such applications represent a very critical issue as they are usually associated with severe diseases. For these reasons several research activities were carried out in recent years with the aim of studying the effects of stress and/or thermally induced phase transformations on fatigue^5–13^ and fracture properties of SMAs^14–38^, in terms of crack formation and propagation mechanisms. For this purpose, special *ad-hoc* investigation techniques have been recently applied to analyze near crack tip transformations, such as synchrotron X-Ray micro-Diffraction (XRD)^14–16^, Infrared thermography (IR)^17,18^, Digital Image Correlation (DIC)^18–22^ and instrumented nano-indentation^23^. In particular, both strain field and lattice evolutions have been analyzed by XRD. These studies have revealed the occurrence of stress-induced transformation (B2-B19′) in austenitic alloys and detwinning/reorientation of B19′ structure in martensitic ones. Similar results have been obtained by Infrared (IR) thermography. In fact, IR investigations have shown the occurrence of both direct (B2-B19′) and reverse (B19′-B2) crack tip transformations under fatigue loadings, thanks to the latent heat associated with stress-induced transformations. In addition, displacement and strain fields in near crack tip regions have been analyzed by DIC. More recently, DIC data have also been used to estimate the effective Stress Intensity Factor (SIF)^20–22^. In particular, the effective SIF has been obtained from a numerical fitting of the measured displacement field by the William’s series expansion^24^. The knowledge of the effective SIF is of great importance since it allows defining both fast fracture conditions and stable fatigue crack growth. In fact, fracture mechanics based approaches have been largely used for fatigue investigations, *i.e.* in terms of crack growth rates (d*a*/d*N*) and fatigue threshold (ΔK_th_), as reported in some recent experimental works^5–8,20^. Finally, more recently, crack tip transformation mechanisms have been analyzed by local mechanical measurements based on instrumented indentation^23^. In particular, stress-induced martensitic transformations have been captured by the indentation response and the effects of the testing temperature have been also analyzed. All these experimental studies, have confirmed that highly localized stresses arising in the crack tip region cause local stress-induced texture evolutions, leading to the formation of detwinned martensite at the very crack tip. These phase transitions play a significant role on crack evolution mechanisms under both static and fatigue loading conditions.

To better understand the effects crack tip texture evolutions on fracture and fatigue properties of SMAs, numerical and analytical studies have been carried out. In particular, the Finite Element (FE) method, with special constitutive models for SMAs, has been used to analyze the near crack tip stresses and transformation mechanisms^25–32^. In addition, some analytical models have been developed^33–39^, which are mainly based on modified linear elastic fracture mechanics (LEFM) concepts. In particular, a novel analytical method has been developed in ref. 36, based on a modified Irwin’s correction of the LEFM^24^. This method allows predicting the extent of crack tip transformation region as well as the resulting stress distribution. Furthermore, based on this model fracture control parameters for SMAs have been proposed^39^, *i.e.* special SIFs which account for the crack tip transformation and the actual stress distribution. However, despite the large number of research reports on fracture of NiTi SMAs, much effort should be paid for an effective understanding of the role of phase transformations on the crack formation and propagation mechanisms.

Within this context, systematic experiments and theoretical studies were carried out in this investigation with the aim of capturing the actual stress-strain distribution at the crack tip. The effects of temperature on fracture properties of SMAs, within the pseudoelastic regime of the alloys, were also analyzed. In particular, temperature controlled fracture tests were carried out, by using single edge crack specimens made of a commercial pseudoelastic NiTi alloy. The DIC method was applied to capture displacement and strain distribution in the crack tip region. In addition, the effective SIF was estimated from a numerical fitting of the measured displacement field by the William’s series expansion. Furthermore, the experimental results were critically analyzed by using a recent analytical model^37^ and it was demonstrated that this model is able to correctly capture the effects of temperature on the crack tip stress distribution. As a consequence, the model can be actually used to define an effective fracture toughness parameter for SMAs by taking into account the real thermo-mechanical loading conditions, including both mechanical load and temperature, and the resulting stress-induced transformation phenomena.

## Materials and Methods

### Material and specimen

**Figure 1:**
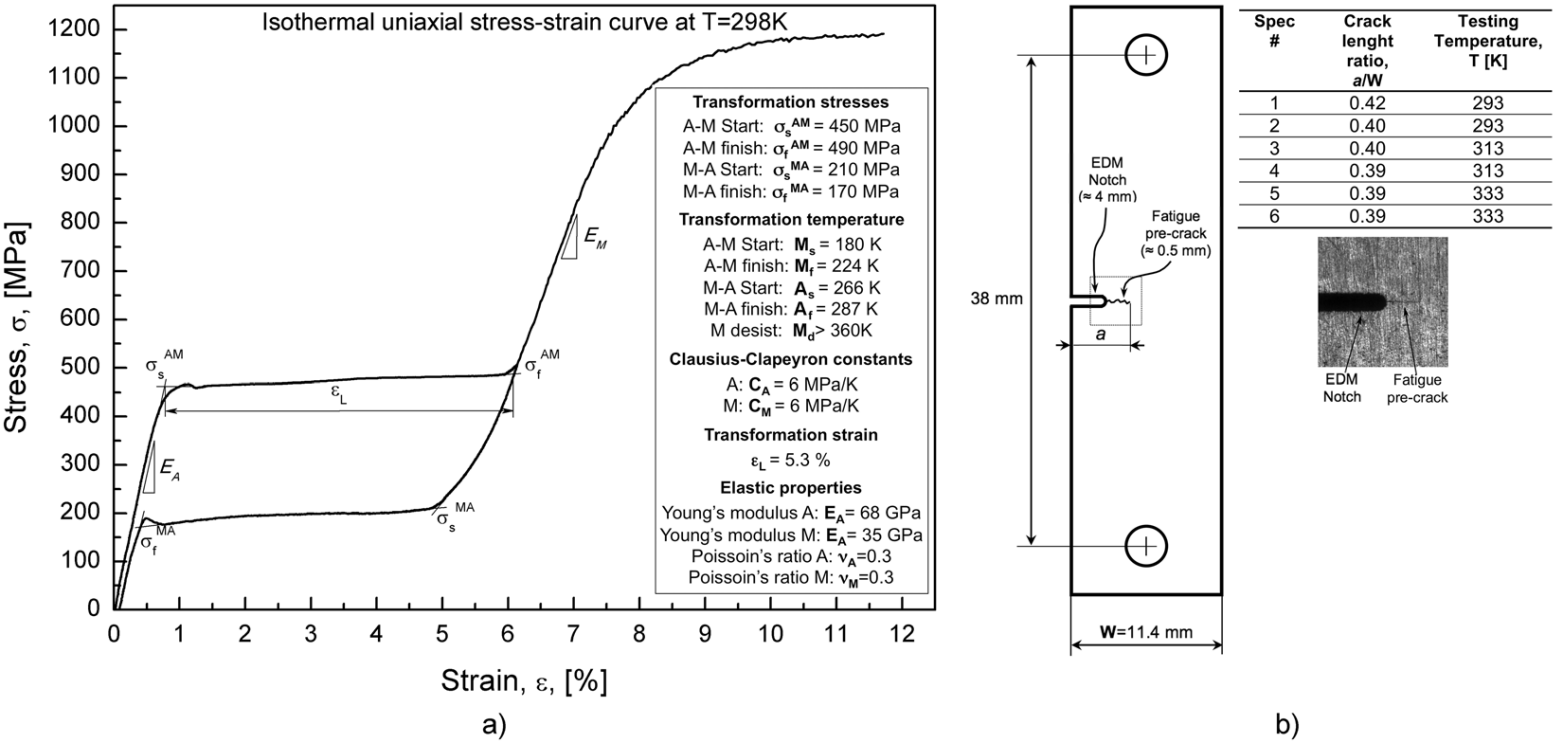
(**a**) Isothermal stress-strain response of the alloy (T = 298 K) together with the main thermo-mechanical parameters; (**b**) Geometry and dimensions of the Single Edge Crack (SEC) specimen used for fracture tests with an highlight of the crack tip region.

A commercial Ni rich NiTi alloy (50.8 at.% Ni–49.2 at.% Ti), was analyzed in this investigation. Figure 1a illustrates the isothermal (T = 298 K) stress-strain response of the alloy together with the measured values of the main thermo-mechanical parameters, namely transformation stresses (${\sigma }_{s}^{AM}$, ${\sigma }_{f}^{AM}$, ${\sigma }_{s}^{MA}$ ${\sigma }_{f}^{MA}$), transformation temperatures (*M*_*s*_, *M*_*f*_, *A*_*s*_, *A*_*f*_), martensite desist temperature (M_d_), transformation strain (*ε*_*L*_), Young’s moduli (*E*_*A*_, *E*_*M*_), Poisson’s ratios (ν_A_, ν_M_) and Clausius-Clapeyron constants (*C*_*A*_, *C*_*M*_).

Single Edge Crack (SEC) specimens, with dimensions shown in Fig. 1b, were manufactured from NiTi sheets with thickness *t* = 0.5 mm, by Electro Discharge Machining (EDM). The rolling direction is parallel to the tensile axis. The samples were fatigue pre-cracked (*f* = 5 Hz, R = *σ*_*min*_/*σ*_*max*_ = 0 and *σ*_*max*_ = 20 MPa), starting from EDM notch (radius around 100 μm), up to a length to width ratio, *a/W*, close to 0.40. Almost straight crack paths normal to the load direction, initiating from EDM notches, were obtained in all specimens as illustrated in the optical micrographs of Fig. 1b.

Isothermal displacement controlled (0.05 mm min^−1^) fracture tests were carried out by monotonic tensile loading until fracture. The tests were executed at different temperatures within the pseudoelastic regime of the alloy, *i.e.* A_f_ < T < M_d_ (T_1_ = 293 K, T_2_ = 313 K and T_3_ = 333 K). Two specimens for each testing temperature were analyzed, as illustrated in Fig. 1b.

Mechanical tests were carried out by an electro-dynamic testing machine (Instron E10000) equipped with and a special system for open-air temperature control. In this system heat is provided by a Peltier cell, which is directly applied on one side of the specimen. A K-type thermocouple and an electronic control driver unit is used for feed forward temperature control, with an accuracy of 0.5 °C. Direct measurements and control of the specimen temperature allows to avoid possible temperature variations arising during tensile test, due to the latent heat associated with stress-induced transformations. Crack propagation and evolution was monitored *in-situ* during mechanical tests by using a CCD Camera (Sony ICX 625 – Prosilica GT 2450) with a resolution of 2448 × 2050 pixels. A suitable objective was adopted to focus the crack tip region (Rodagon f. 80 mm – Rodenstock), which allows obtaining a resolution of approximately 360 pixels/mm. Finally, Digital Image Correlation was performed, by using a commercial DIC software (VIC-2D^®^, Correlated Solutions), to capture the near crack tip displacement and strain fields.

### Fracture mechanics in SMAs: basics mechanisms and analytical modeling

In this section, a basic description of the crack tip mechanisms in SMAs together with a summary of the analytical model described in ref. 37 are reported for the sake of completeness.

**Figure 2:**
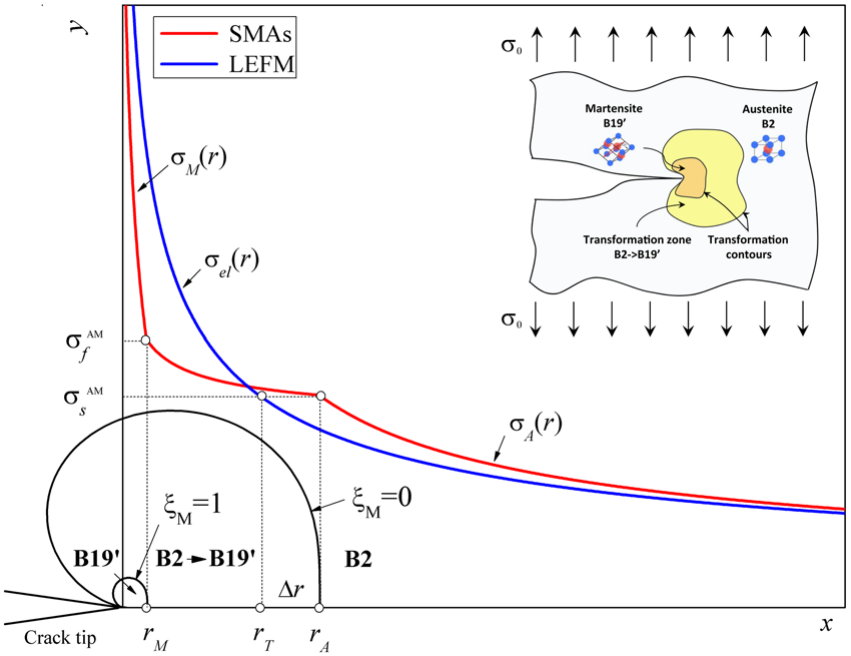
Schematic depiction of the crack tip stress distribution and transformation region in SMAs.

As illustrated in Fig. 2, stress-induced phase transformations in SMAs cause a complex crack tip stress distribution, if compared to common engineering metals. This distribution results from the large strain associated with SIM as well as from the intrinsic different elastic properties of the two crystallographic phases (B2 and B19′). In fact, three different regions are observed near the crack tip: (1) fully transformed martensitic zone at the very crack tip (*r* < *r*_*M*_) with martensite volume fraction ξ_M_ = 1, (2) transformation region (*r*_*M*_ < *r* < *r*_*A*_) with 0 < ξ_M_ < 1 and (3) austenitic untransformed region (*r* > *r*_*A*_) where ξ_M_ = 0. As a consequence, well known fracture mechanics theories and standard procedures, based on linear elastic (LEFM) or elastic plastic fracture mechanics (EPFM), cannot be directly applied to SMAs.

To this aim, an *ad-hoc* analytical approach^37^ was used in this investigation, which is based on modified LEFM relations and on the assumption of small scale transformation^39^. This method allows predicting both the crack tip transformation mechanisms and the resulting stress and strain distributions. The crack tip fields are conveniently expressed in terms of polar coordinates (*r*, *θ*) with origin at the crack tip. In particular, under mode I loading and for *θ* = 0 the principal stress components in the austenitic untransformed region, *σ*_*Ai*_(*r*), are given by a modified Irwin correction of the LEFM, *i.e.* by using effective crack length and stress intensity factor, *a*_*e*_ and *K*_*Ie*_ respectively:

1 $${\sigma }_{Ai}(r)={g}_{i}\frac{{K}_{Ie}}{\sqrt{2\pi (r-{\rm{\Delta }}r)}}$$

where *g*_*i*_ = 1 for *i* = 1, 2 and *g*_*i*_ = *b* for *i* = 3, with *b* = 0 for plane stress and *b* = 2ν for plane strain (Poisson’s ratio ν = ν_A_ = ν_M_); Δ*r*, *a*_*e*_ and *K*_*Ie*_ are given by (see Fig. 2):

2 $${\rm{\Delta }}r={r}_{A}-{r}_{T}$$

3 $${a}_{e}=a+{\rm{\Delta }}r$$

4 $${K}_{Ie}=\beta ({a}_{e}/W){\sigma }_{0}\sqrt{\pi {a}_{e}}$$

where *β*(*a*_*e*_/*W*) for SEC specimens^23^ and the radius *r*_*T*_ are given by:

5 $$\beta (\frac{{a}_{e}}{W})=1.12-0.231(\frac{{a}_{e}}{W})+10.55{(\frac{{a}_{e}}{W})}^{2}-21.72{(\frac{{a}_{e}}{W})}^{3}+30.39{(\frac{{a}_{e}}{W})}^{4}$$

6 $${r}_{T}=\frac{{(1-b)}^{2}}{2\pi }{(\frac{{K}_{I}}{{\sigma }_{s}^{AM}})}^{2}$$

The principal stress components for θ = 0 in the fully transformed martensitic region, *σ*_*Mi*_(*r*), are obtained by compatibility conditions and can be expressed as:

7 $${\sigma }_{Mi}(r)={g}_{i}\frac{2(1-\nu -b\nu ){K}_{Ie}/\sqrt{2\pi r}-{E}_{A}{\varepsilon }_{L}+{\alpha }_{M}^{-1}{\sigma }_{f}^{AM}-{\sigma }_{s}^{AM}}{(1-b){\alpha }_{M}^{-1}+(b+1)(1-2\nu )}$$

where *α*_*M*_ = *E*_*M*_/*E*_*A*_ is the Young’s modulus ratio. The transformation radii, *r*_*M*_ and *r*_*A*_, are given by the following equations:

8 $${r}_{M}=\frac{2}{\pi }{(\frac{(1-\nu -b\nu )(1-b){K}_{Ie}}{(1-b){E}_{A}{\varepsilon }_{L}+(b+1)(1-2\nu ){\sigma }_{f}^{AM}+(1-b){\sigma }_{s}^{AM}})}^{2}$$

9 $${r}_{A}=\frac{2(1-b){K}_{Ie}^{2}}{\pi {\sigma }_{s}^{AM}({\sigma }_{s}^{AM}+{\sigma }_{f}^{AM})}+\frac{2({E}_{A}{\varepsilon }_{L}+{\alpha }_{M}^{-1}{\sigma }_{f}^{AM}-{\sigma }_{s}^{AM}){r}_{M}+4(1-\nu -b\nu ){K}_{Ie}\sqrt{2{r}_{M}/\pi }}{(1-b){\alpha }_{M}^{-1}+(b+1)(1-2\nu )({\sigma }_{s}^{AM}+{\sigma }_{f}^{AM})}+{r}_{M}$$

Finally, starting from the stress equations (1) and (7) two different stress intensity factors can be defined, in the austenitic and martensitic regions. In particular, the mode I austenitic SIF, namely *K*_*IA*_, can be directly obtained from Eq. (1) by considering the distance from the effective crack tip ($\tilde{r}=r-{\rm{\Delta }}r$), according to the Irwin’s assumption:

10 $${K}_{IA}=\mathop{\mathrm{lim}}\limits_{\tilde{r}\to 0}\sqrt{2\pi \tilde{r}}{\sigma }_{A}={K}_{Ie}$$

The mode I martensitic SIF, namely *K*_*IM*_, can be obtained from Eq. (7):

11 $${K}_{IM}=\mathop{\mathrm{lim}}\limits_{r\to 0}\sqrt{2\pi r}{\sigma }_{M}=\frac{2(1-\nu -b\nu )}{(1-b){\alpha }_{M}^{-1}+(b+1)(1-2\nu )}{K}_{Ie}$$

Equation (11) shows that *K*_*IM*_ can be expressed as a function of *K*_*Ie*_ by a material constant coefficient. However, it is worth noting that the knowledge of the extent of transformation region, in terms of both *r*_*M*_ and *r*_*A*_, is required to calculate *K*_*IA*_ and *K*_*IM*_ by an iterative approach, similarly to the Irwin’s correction for elastic-plastic materials.

### Digital Image Correlation Measurements

Digital Image Correlation method allows direct measurement of near crack tip displacement and strain fields. In addition, the effective stress intensity factor was estimated by a numerical fitting of displacement data. In particular, the analytical near crack tip displacement field under mode I loading, according to LEFM theory, can be expressed by the William’s expansion series in terms of polar coordinates (*r*, *θ*):

12 $${u}_{x}(r,\theta )=\frac{{K}_{I}}{\mu }\sqrt{(\frac{r}{2\pi })}\,\cos (\frac{\theta }{2})[\frac{1}{2}(k-1)+{\sin }^{2}(\frac{\theta }{2})]+\frac{1}{2\mu }\frac{1}{(1+v)}Tr\,\cos (\theta )-Ar\,\sin (\theta )+{B}_{ux}$$

13 $${u}_{y}(r,\theta )=\frac{{K}_{I}}{\mu }\sqrt{(\frac{r}{2\pi })}\,\sin (\frac{\theta }{2})[\frac{1}{2}(k+1)-{\cos }^{2}(\frac{\theta }{2})]-\frac{1}{2\mu }\frac{v}{(1+v)}Tr\,\sin (\theta )+Ar\,\cos (\theta )+{B}_{uy}$$

where *μ* = *E*/[2(1 + *v*)] is the shear modulus of elasticity; *T* is the *T*-stress parameter; A and B represent the rigid body motions (B_ux_ and B_uy_) and rotations (A). The second order term in the William’s expansion series, *i.e.* the *T*-stress parameter, is included due to the large fracture process zone in SMAs, associated with near crack tip stress-induced transformations^22^. In fact, as well known, the size of the investigation windows should be larger than the K-dominant zone and, consequently, *T*-stress term becomes a non-negligible parameter. In particular, based on preliminary studies^22^ a fitting region of about 5 mm × 4 mm around the crack tip was used.

**Figure 3:**
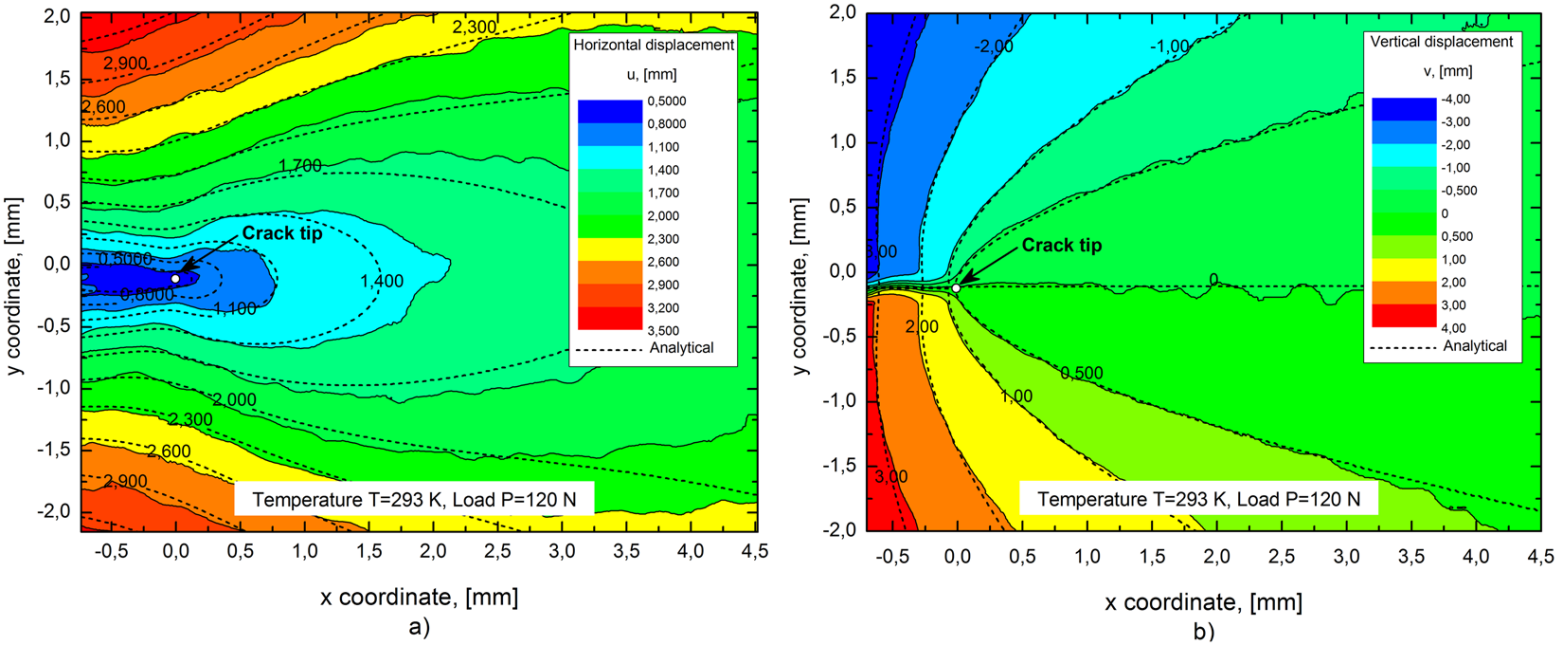
DIC displacement contours *vs* analytical solution (black dashed curves) for a SEC specimen (*a/W* = 0.43) at *P* = 120 N and *T* = 293 K: (**a**) horizontal displacement *u*_*x*_ and (**b**) vertical displacement *u*_*y*_.

Starting from DIC displacement components, namely *u*_*x0*_ and *u*_*y0*_, an over-deterministic approach and the linear least square regression method can be used to estimate the stress intensity factor (K_I_) and the other parameters in eqs 12 and 13 (T, A, and B). The method is capable of providing well estimations of the unknown parameters with low calculation error, due to the very high number of equations compared to the unknowns. However, when crack tip transformation region becomes not negligible with respect to K-dominant zone, LEFM is no longer valid. As a consequence an effective stress intensity factor (K_Ie_) can be calculated, in order to take into account the crack tip non-linearity, such as by using the Irwin’s correction method described in section 2.2 (see eq. 1). In particular, an effective crack length (*a*_e_) can be considered (see eq. 3), *i.e.* the origin of the coordinate systems of eqs. 12 and 13 has to be located at the effective tip. It can be done by moving the reference system along the θ = 0 direction by a distance Δr (see eq. 2). To this aim the following coordinate substitution is carried out:

14 $$\theta ^{\prime} ={\tan }^{-1}(\frac{r\,\sin \,\theta }{r\,\cos \,\theta -{\rm{\Delta }}r})$$

15 $$r^{\prime} =\sqrt{{r}^{2}+{\rm{\Delta }}r-2r{\rm{\Delta }}r\,\cos \,\theta }$$

the distance Δ*r* between the real and the effective crack tip is obtained from data fitting. As an example Fig. 3 shows a comparison between the analytical displacements, described by equations (12 and 13), and the experimental ones, obtained by means of the digital image correlation. In particular, the figures illustrate the contour plots of u_x_ (Fig. 3a) and u_y_ (Fig. 3b) in the SEC specimen #1 (*a*/W = 0.43) under mode I loading at P = 120 N (σ_0_ = 20 MPa) and T = 293 K. The comparison shows good agreement between experimental data and analytical results even at large distance from the crack tip (around 4 mm) thanks to the use of the T stress term in eqs. 12 and 13, as discussed in ref. 22.

## Results and Discussions

### Crack-tip strain and phase transitions

**Figure 4:**
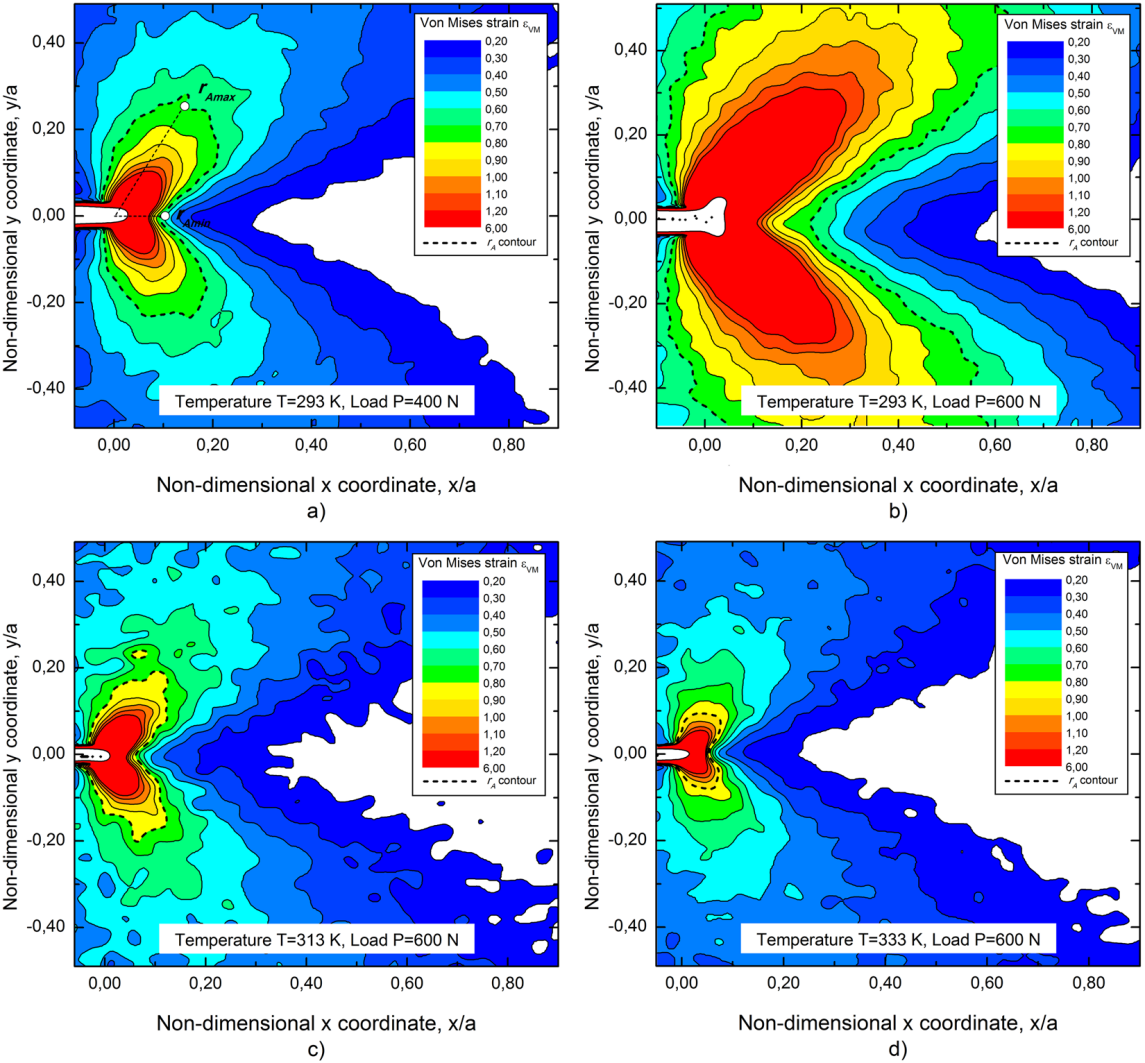
DIC strain contours obtained from SEC specimens at different thermo mechanical loading condition: (**a**) P = 400 N and T = 293 K; (**b**) P = 600 N and T = 293 K; (**c**) P = 600 N and T = 313 K; (**d**) P = 600 N and T = 333 K.

Figure 4 illustrates the near crack tip von Mises strain contours obtained from DIC measurements under different thermo-mechanical loading conditions. In particular, Fig. 4a,b show the strain distribution measured at *T* = 293 K on SEC sample #1 (see Fig. 1b) at *P* = 400 N (σ_0_ = 70.2 MPa) and at *P* = 600 N (σ_0_ = 105.3 MPa), respectively. Figure 4c,d illustrate the results at *T* = 313 K and *P* = 600 N on SEC #3 (Fig. 4c) and at *T* = 333 K and *P* = 600 N on SEC #5 (Fig. 4d).

All figures show a butterfly-like shape, which are very similar to common elastic plastic metals, and the black dashed contours denote the approximate extension of the transformation region (*i.e. r* < *r*_*A*_ in Fig. 2). In particular, the transformation contours (*r*_*A*_) are identified by the values of the von Mises strain, *i.e.* by using the temperature dependent strain value corresponding to the onset of the stress-induced martensitic transformation. As clearly shown in Fig. 4b the maximum extent of the transformation region, namely *r*_*Amax*_, is observed at an angle *θ* around 60°, while the minimum one, namely *r*_*Amin*_, is observed at θ = 0. This result is similar to the plastic region in common elastic plastic materials^40^. In addition, the fully transformed zone (*r* < *r*_*M*_) cannot be identified by the adopted experimental setup due to the very small size with respect to the observation window. However, the extent of the fully transformed region (*r*_*M*_) is about two order of magnitude smaller than that of the transformation region (*r*_*A*_) and, therefore, it can be neglected when computing the stress intensity factor.

The comparison between Fig. 4a (P = 400 N) and 4b (P = 600 N) shows, as expected, a marked effect of the applied load on the extent of the transformation region (*r*_*A*_). In fact, *r*_*A*_ is proportional to *K*_*I*_^2^ (see eqs. 8 and 9). This implies a marked variation of the effective SIF (*K*_*Ie*_) with respect to the LEFM prediction (*K*_*I*_) when increasing the applied load (*P*), because *K*_*Ie*_ depends on the effective crack length (*a*_*e*_) and, consequently, on the transformation radius (see eqs. 2–4). In addition, the comparison between Fig. 4b (T = 293 K), 4c (T = 313 K) and 4d (T = 333 K) shows a significant effect of the testing temperature on the extent of the transformation zone. This result is attributed to the Clausius Clapeyron relation, because *r*_*A*_ is proportional to (1/*σ* ^*AM*^)^2^ (see eqs. 8 and 9). As a consequence, the alloy approaches to a linear elastic behavior when increasing the temperature, due to the associated increase of the transformation stress, *i.e.* effective SIF becomes closer to the linear elastic one. A deeper discussions about SIF in SMAs is reported in the following section.

**Figure 5:**
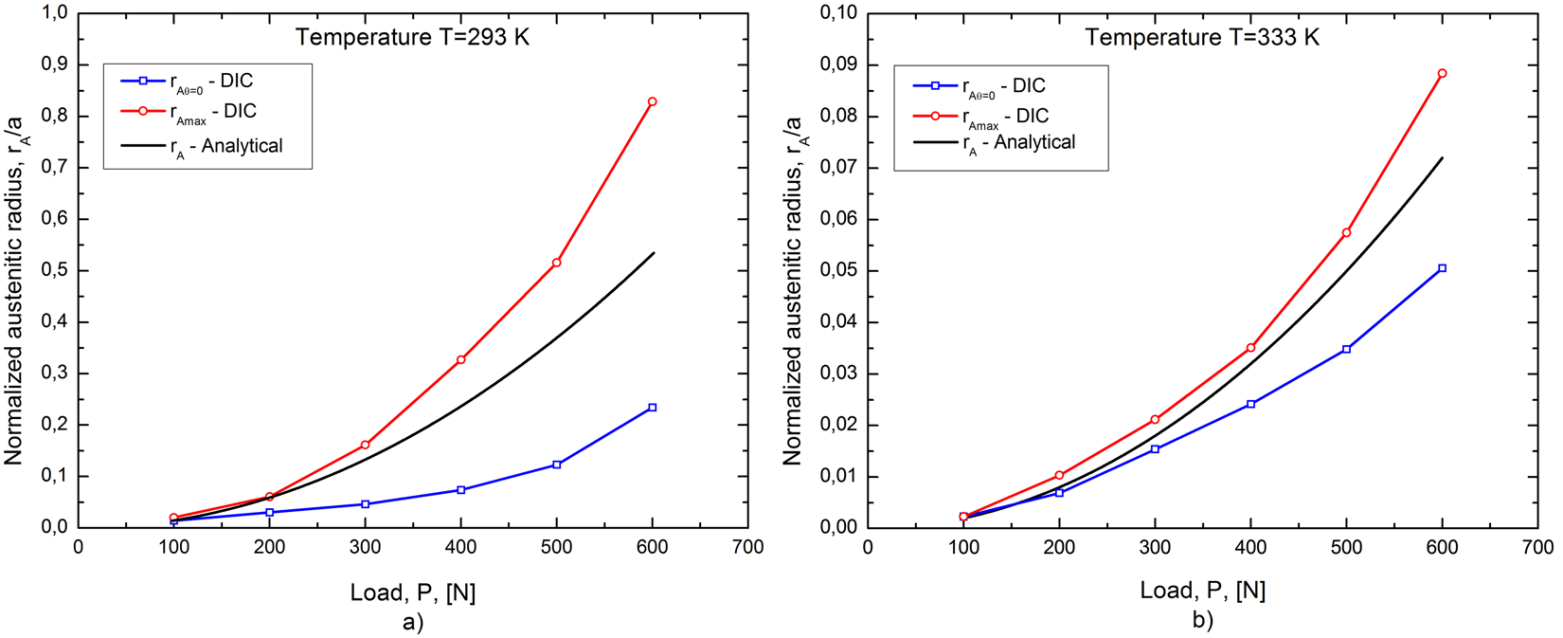
Comparison of the normalized austenitic radius *vs* load (*r*_A_/*a vs P*), between analytical model and DIC results: (**a**) *T* = 293 K and (**b**) T = 333 K.

Figure 5 illustrate comparisons between the predictions of the analytical model and the DIC results in terms of the austenitic radius *r*_*A*_. In particular, the evolution of *r*_*A*_/*a* as a function of the applied load *P*, given by eq. 9, is compared with the two bounds (*r*_*Amin*_ and *r*_*Amax*_) obtained from DIC at the at the testing temperature *T* = 293 K (Fig. 5a) and at *T* = 333 K (Fig. 5b). As expected, both figures show that the analytical results are always between the lower (*r*_*Amin*_) and upper (*r*_*Amax*_) limits obtained from DIC. In fact, the analytical model is based on the *θ* = 0 assumption and does not consider the complex multiaxial stress-strain redistribution occurring at different angles as a consequence of stress induced transformations.

### Stress Intensity Factor and fracture toughness measurements

**Figure 6:**
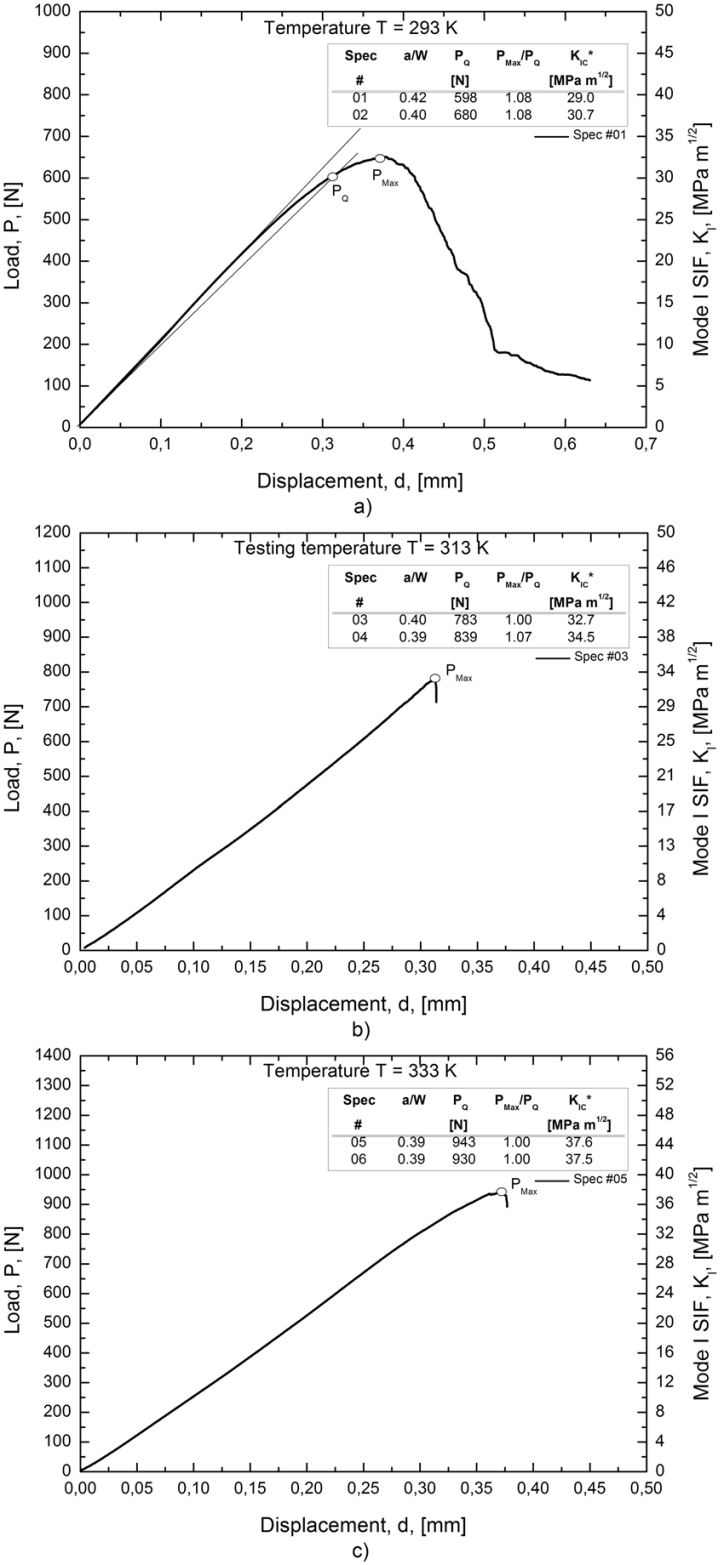
Laod (*P*) and Stress intensity factor (*K*_*I*_) *vs* displacement (*d*) curves obtained from fracture tests of SEC specimens at three different values of the testing temperature: (**a**) T = 293 K (sample #1); (**b**) T = 313 K (sample #3) and (**c**) T = 333 K (sample #5).

Figure 6 illustrate load-displacement (*P*-*d*) curves obtained from fracture tests carried out at the three different values of the testing temperature (*T*_*1*_ = 293 K, *T*_*2*_ = 313 K and *T*_*3*_ = 333 K). The values of the mode I stress intensity factors (*K*_*I*_) can be also obtained from the right vertical axis of the figures, as the ratio *K*_*I*_ /*P* is a constant depending on the *a*/*W* ratio of the specimen. In addition, relevant data obtained from all tested specimens (*a*/*W* ratio, *P*_*max*_ and *P*_*Q*_) are shown in the figures. In particular, the load *P*_*Q*_ was calculated according to the standard ASTM E399 (see Fig. 6a) and it was used for fracture toughness calculations, *i.e.* for the critical value of the stress intensity factor, namely *K*_IC_*. It is worth noting that, even though most of the conditions given by ASTM standards are not satisfied in the case of SMAs, due to several material/specimen limitations, Fig. 6 show that *P-d* curves exhibit an almost linear trend in the early stage of loading with limited nonlinear deviations near the maximum load (*P*_*max*_). In fact, these nonlinearities, which are more evident for the lower values of the testing temperature, are always within the acceptability range of the standard ASTM E399 (*P*_*max*_/*P*_*Q*_ < 1.1) as shown in Fig. 6.

In addition, the figures also show that fast fracture always occurs at *T*_*2*_ = 313 K and *T*_*3*_ = 333 K, while stable crack propagations was observed in the specimens tested at *T*_*1*_ = 293 K. This is evident from a softening of the *P-d* curve after *P*_*max*_, similarly to the results reported in ref. 16. It is in agreement with the temperature dependent stress-strain relation in SMAs. In fact, material non linearity decrease with increasing the temperature, according to the Clausius-Clapeiron relation, *i.e.* transformation stresses rise and the alloy tends to approach a linear elastic behavior. However, a unusual trend was observed with increasing the testing temperature, because a systematic increase of both *P*_*max*_ and *P*_*Q*_ were recorded. This trend is exactly the opposite one could expect from standard metals, where material nonlinearity associated with large plasticity usually has a toughening effect, *i.e.* it cause an increase in the fracture strength of the material.

**Figure 7:**
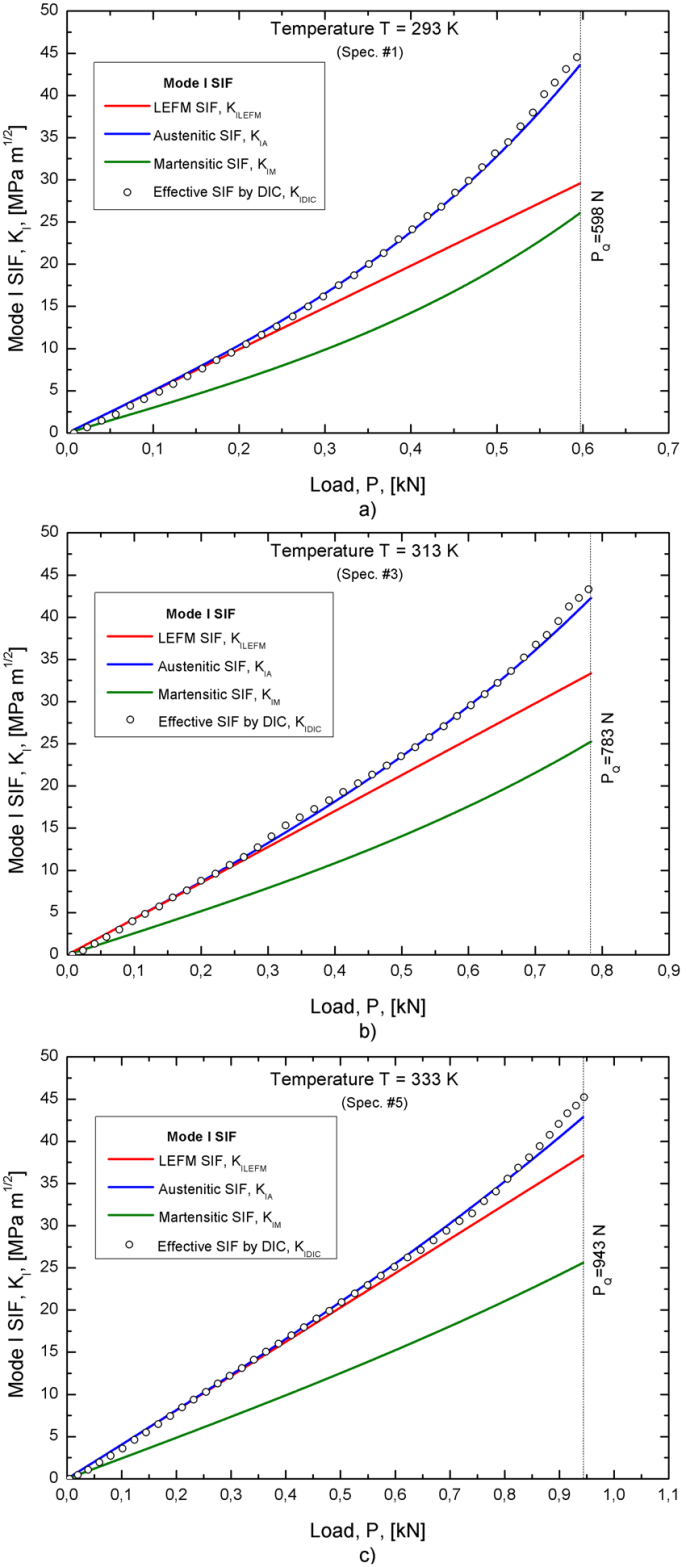
Stress intensity factor *vs* load curves (*K*_*I*_ vs *P*) obtained from fracture tests of SEC specimens at three different values of the testing temperature: (**a**) T = 293 K (sample #1); (**b**) T = 313 K (sample #3) and (**c**) T = 333 K (sample #5).

To better understand this phenomenon, stress intensity factors obtained from different methods have been systematically compared, as shown in Fig. 7. In particular, SIF obtained from linear elastic fracture mechanics theory, namely *K*_*ILEFM*_, are plotted together with the austenitic and martensitic SIFs obtained from the analytical model, namely K_IA_ and K_IM_ (eqs. 10 and 11), and with the effective SIF obtained from DIC, namely K_IDIC_ (eqs. 12 and 13). Figure 7a–c reports the curves *SIF vs* applied load (*K*_*I*_ *vs P*) for the testing temperatures *T*_*1*_, *T*_*2*_ and *T*_*3*_, respectively, *i.e.* for samples #1, #3 and #5, up to the load *P*_*Q*_. In fact, SIF is non longer valid for *P* > *P*_*Q*_ due to marked non linearity’s and/or stable crack growth. All figures show that *K*_*IA*_ and *K*_*IM*_ exhibit a non linear trend and deviations from linear elastic fracture mechanics (*K*_*ILEFM*_) increase when increasing the applied load and when decreasing the testing temperature. In fact, as illustrated and discussed in the previous section (3.1), transformation region and effective crack length rapidly increase with increasing *P* and reducing *T* because they are proportional to K_I_^2^ and to (1/σ^AM^)^2^ (see eqs. 8 and 9). In addition, the figures also show good agreement between the displacement based effective SIF (*K*_*IDIC*_) and the austenitic SIF (*K*_*IA*_). In particular, *K*_*IDIC*_ exhibits the same non-linear trend with maximum differences with respect to *K*_*IA*_ in the near fracture zone never greater than 5%. This is the expected result since DIC data fitting provides the stress distribution in the austenitic untransformed region, as discussed in refs 20–22, because the extent of the fully transformed region is much smaller (two order of magnitude) than the fitting region. Based on these remarkable agreements it can be definitely concluded that the analytical model is able to predict the complex stress-strain distribution in pseudoelastic SMAs under generic thermo-mechanical loading conditions (stress and temperature). This agreement is even more important because the two methods are based on completely different approaches: the analytical model is load-based while the experimental DIC method is displacement-based.

**Figure 8:**
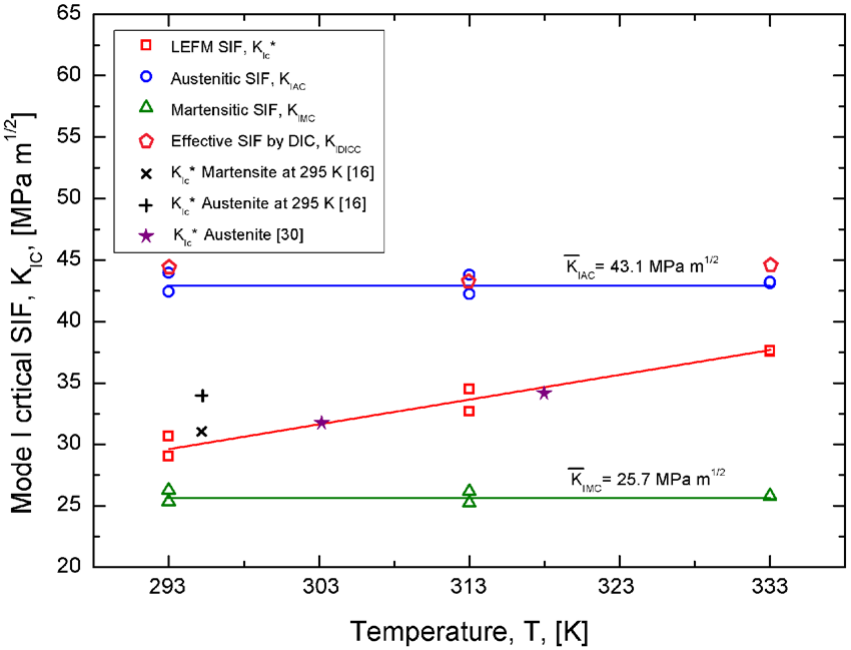
Critical values of the stress intensity factor as a function of the testing temperature: comparison between LEFM, analytical model and DIC results.

Figure 8 reports the critical values of the stress intensity factor, as a function of the testing temperature, obtained from LEFM (*K*_IC_*), DIC (*K*_*IC DIC*_) and analytical model (*K*_*IA C*_ and K_*IM C*_). A systematic increase of *K*_IC_* was observed with increasing the testing temperature, ranging from about 30 MPa m^1/2^ at T = 293 K to about 37.5 MPa m^1/2^ at T = 333 K. However, it is important to point out that this result is limited to the investigated temperature range (293 K–333 K), which corresponds to the practical engineering range for pseudoelastic applications of SMAs (A_f_ < T < M_d_). In any case, Fig. 8 also shows that results obtained in a previous investigations^30^, at different temperature values (303 K and 318 K) within the pseudoelastic regime, are in good agreement with the current results.

The trend of *K*_IC_* with temperature seems to disagree with the literature assumption that stress induced crack tip transformations in SMAs represent a toughening effect. In fact, crack tip transformations become more and more little when increasing the temperature (see Figs 4 and 5) and they completely disappear at the martensite desist temperature (T > M_d_). Even if this is a completely novel result within the SMA community, it is in accordance with systematic experimental results reported in ref. 16 and shown in Fig. 8. In fact, in ref. 16 a marked increase of the critical stress intensity factor is observed at T > M_d_ with respect to pseudoelastic (austenite) and pseudoplastic (martensite) alloys.

On the contrary, the critical values of the austenitic SIF calculated according to the analytical model, *K*_*IA C*_, seems to be almost temperature independent. The same consideration applies to the martensitic SIF, *K*_*IM C*_, because the ratio *K*_*IA*_ */K*_*IM*_ is a material constant (see eq. 11). In addition, the figure shows that the critical values of the effective SIF estimated by DIC, *K*_*IC DIC*_, is very close to the austenitic SIF, as also shown in Fig. 7, and it is temperature independent.

This is a very interesting and novel result, as it is demonstrated that the analytical model is able to correctly capture the effects of complex thermo-mechanical loading conditions in SMAs, *i.e.* in terms of applied stress and temperature. In particular, the increase of the critical SIF, based on LEFM, with increasing the testing temperature cannot be attributed to a change in the material properties at the crack tip, but temperature plays a significant role on the effective crack tip stress-strain distribution. In fact, material properties at the very crack tip are unaffected by the temperature, as also experimentally observed by SEM investigations in ref. 16, *i.e.* crack always grow in the high stress detwinned martensitic phase.

As a consequence, a novel approach should be adopted, with respect to linear elastic or elastic plastic theories. In particular, temperature significantly affects the effective SIF and, consequently, the whole thermo mechanical loading condition has to be taken into account for SIF calculation, i.e. by considering both mechanical load and temperature. In fact, based on our analytical approach a temperature independent critical value of the SIF is obtained, as shown in Fig. 8, and this can be actually considered a material property. The analytical model can be used to define critical conditions for fast fracture in SMAs, *i.e.* by comparing the temperature dependent SIF with its temperature independent critical value.

## Conclusions

Temperature dependent fracture properties of a NiTi-based Shape Memory Alloys (SMAs) were analyzed, within the pseudoelastic regime of the alloy. In particular, the effective Stress Intensity Factor (SIF) was estimated by a fitting of the William’s expansion series, based on Digital Image Correlation (DIC) measurements. In addition, a standard Linear Elastic Fracture Mechanics (LEFM) approach and an *ad-hoc* analytical model for SMAs were applied. The main results can be summarized as follows:

- The temperature plays an important role on fracture properties of SMAs. In fact, it significantly affects the crack tip transformation behavior and the resulting stress-strain distribution. In particular, it was observed that stress-induced phase transitions does not represent a toughening effect. This is a completely novel result within the SMA community, where crack tip stress-induced transformations are considered as toughening effects.
- Large differences were observed between LEFM and DIC results due to the large material nonlinearities in SMAs, resulting from crack tip transformations. In addition, LEFM approaches do not consider the effects of temperature.
- Good agreements were observed between the predictions of the analytical model and the DIC results, in terms of both crack tip transformation region and effective SIF, within the investigated temperature range. In fact, both mechanical load and temperature are taken into account in the analytical model.
- A novel fracture toughness parameter for SMA can be defined, based on the analytical model, which can be actually considered as a temperature independent material property. Therefore, a new fracture mechanics approach for pseudoelatic SMAs is proposed, where both mechanical load and temperature is taken into account for SIF computation.
